# Accurate translation is important for longevity

**DOI:** 10.18632/aging.101398

**Published:** 2018-03-12

**Authors:** Zhonghe Ke, Andrei Seluanov, Vera Gorbunova

**Affiliations:** 1 University of Rochester, Department of Biology, Rochester, NY 14627, USA

**Keywords:** longevity, aging, protein translation, translation fidelity

When a protein is made by the cell, the genetic information is first decoded into mRNA, then this mRNA directs the protein synthesis. This is the flow of genetic information in cells from DNA to RNA to protein, the central dogma of molecular biology. The “error catastrophe” theory of aging, proposed by Orgel in the 1960s [1], posits that translation errors decrease the fidelity of translation, setting in motion a vicious cycle of increasingly inaccurate protein synthesis, ultimately causing a failure of the gene expression machinery. However, in the 1980s, several approaches including enzymatic assays of protein synthesis errors, analysis of proteins on 2D gels in aged animals and senescent cells did not detect a significant increase in mistranslated proteins during aging and cellular senescence. These negative results were not consistent with the error catastrophe theory and errors in translation were largely discounted as being a contributing factor to aging.

A recent report by Ke et al. [2] brings the protein translation fidelity back into the spotlight of aging research. Importantly, the assays used to detect aberrant proteins in the 1980s had limited sensitivity to detect rare aberrant proteins. Errors in translation are estimated to occur every 2,000-10,000 amino acids. Under normal conditions, mistranslated proteins are present in cells at a negligible level.

In 2013, Azpurua et al. reported a new highly sensitive luciferase-based assay to measure the rate of mistranslation in mammalian cells [3]. This new assay detects mistranslation events leading to re-activation of a mutated luciferase protein. Using this assay, Azpurua et al. showed that mouse fibroblasts make up to 10 times more errors in protein translation than fibroblast from the longest-lived rodent species, the naked mole rat. This was the first indication that a longer-lived species may evolve more accurate protein translation machinery. Ke et al. [2] expanded this study and compared the fidelity of protein translation in fibroblasts from 17 rodent species with diverse lifespans. The study demonstrated that translation fidelity at the first and second codon positions correlates positively with species maximum lifespan [2], i.e. longer-lived species have more accurate translation. This study shows that the basal rate of translation errors is important in defining species lifespan.

The relationship between species maximum lifespan and translation fidelity shows that longer-lived species evolve more accurate protein synthesis. This, however, does not imply that protein translation errors lead to aging in individual organisms. This would be important to test using the new sensitive assays. In the future, a knock-in mouse model with luciferase reporters can be generated to examine the accumulation of mistranslated protein in different organs during aging.

Mistranslated proteins may not impact cellular proteostasis significantly at young age, largely due to rapid protein turnover and efficient protein clearance. However, protein turnover rates, proteasome activity, and autophagy decline with age [4–6], making aged organisms more sensitive to errors in protein translation. Thus, even if protein translation fidelity does not change over the course of lifespan, long-lived species may require more accurate protein synthesis.

**Figure 1:**
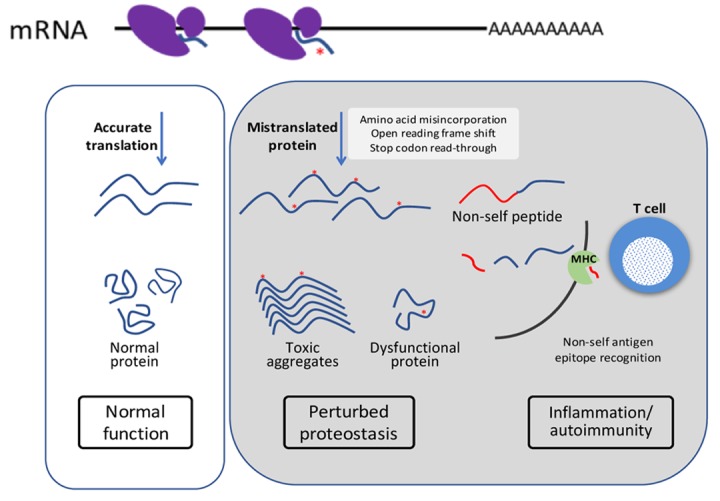
Errors in protein translation lead to perturbed proteostasis, protein aggregates and inflammation.

Aberrant proteins are associated with a variety of disease conditions and can hurt the organism in several ways (Figure 1). Age-associated neurodegenerative diseases, such as Alzheimer’s (AD), Parkinson’s (PD), and Huntinton’s disease (HD) are caused by accumulation of protein aggregates. The initial aberrant proteins that act as a seed for protein aggregates may originate from errors of protein translation machinery. Some of the mistranslated proteins aggregate and become infectious, and then change the conformation of normal proteins and propagate the aggregates. The accuracy of protein translation is controlled at multiple levels, one of which is aminoacyl-tRNA synthetases. In humans, 21 aminoacyl-tRNA synthetases are responsible for putting cognate amino acid to tRNA accurately and crucial for translation fidelity. Mutations in glycyl-tRNA synthetase, tyrosyl-tRNA synthetase, lysyl- tRNA synthetase and alanyl- tRNA synthetase have been shown to be associated with Charcot-Marie-Tooth (CMT) disease, one of the most common inherited neurological disorders [7]. Given that, translation fidelity is very crucial for maintaining proteostasis. Mistranslated proteins may also pose a risk of autoimmunity. Mistranslated peptides, can be presented on the cell surface by MHC complex, and recognized as non-self antigens. Chronic sterile inflammation is a hallmark of aging, and errors in protein synthesis may accelerate the onset of autoimmunity. Thus, although errors in protein translation are rare, they may trigger the onset of multiple age-related pathologies.

In summary, the new findings by Ke et al. [2] show that long-lived species evolve more accurate protein translation machineries. This finding revives the connection between translation fidelity and aging. Whether translation fidelity declines with age remains to be tested. However, even if the basal rate of translation errors does not change during aging as was proposed by the original error catastrophe theory, the time when organismal function starts to decline and age-related pathology sets in may be determined by the fidelity of protein synthesis. Species with higher translation fidelity are expected to maintain proteostasis for longer time. In conclusion, the new studies suggest translation fidelity plays an important role in organismal longevity.
